# Notes and Notices

**Published:** 1891-07

**Authors:** 


### NOTES AND NOTICES.

The Indiana Institute of Homœopathy held its twenty-fifth annual session May 13th, at the State House, Indianapolis, with prayer by the Rev. D. R. Lucas. A rousing address of welcome was delivered by Dr. O. S. Runnells, of Indianapolis, responded to by President E. W. Sawyer, of Kokomo.

The Secretary, Dr. W. B. Clarke, of Indianapolis, read minutes of the last meeting, and the Treasurer, Dr. J. S. Martin, of Muncie, made his report. Among the distinguised visitors from other States were: Prof. H. B. Fellows, Dr. J. B. S. King, Hahnemann College, Chicago; Professor Jewett, of the Cleveland College, all of whom made addresses. Among those present from the State were: S. C. S. Fahnestock, Laporte; E. C. Cole, Michigan City; M. H. Waters, W. H. Baker, Terre Haute; F. H. Huron, Danville; W. B. Huron, Tipton; W. R. Rently, Morristown; E. A. Edmonds.

President Sawyer, in his address, after congratulating the members upon the increased growth of the Society, spoke of the fact that a large proportion of the wealthy and intelligent of Indiana were believers in and patrons of Homœopathy, but there was not a public institution in the State that was controlled by homœopathists. He thought if Homœopathy got a fair chance and an equal standing before the law it would have nothing to fear, even in the centre of insane hospitals, as has been abundantly shown in other States which have tried it, notably New York, Illinois, Massachusetts, and Minnesota. The unfairness of the prevailing plan of a State examining board was alluded to as being especially unjust to the schools that are numerically weakest. He briefly reviewed the progress of medicine during the year, the comparisons made being decidedly in favor of Homœopathy.

In the two days' session a large number of papers were read and discussions followed, after which there was an election of officers for the ensuing year which resulted as follows: President, J. T. Boyd, Indianapolis; Vice-President, E. Z. Cole, Michigan City; Second Vice-President, J. H. Allen, Logansport; Treasurer, J. S. Martin, Muncie; Secretary, W. B. Clarke, Indianapolis. Drs. Martin and Clarke were re-elected.

According to the *Indianapolis Sentinel*, to which we are indebted for this report, Dr. Sawyer made a capable President, Dr. King a full stenographic report, Treasurer Martin a satisfactory financial exhibit, and Secretary Clarke, a hard-working officer.

Minnesota State Homœopathic Institute have elected the following officers for the ensuing year: President, Dr. D. A. Strickler, St. Paul; First Vice-President, T. W. Ashley, River Falls; Second Vice-President, George T. Robinson, Minneapolis; Secretary, E. W. Honning, of Minneapolis; Treasurer, D. A. Locke, Lake City. Delegates to American Institute of Homœopathy, W. S. Briggs, J. E. Sawyer, Alexander Donald, St. Paul; A. E. Higbee, Minneapolis; O. H. Hull, Zumbrota. Delegates to the Wisconsin State Society, Charles Pillsbury, Duluth; and P. Roberts, Winona. The Censors are: L. M. Spaulding and H. W. Brazee, Minneapolis; and C. H. Glidden, St. Paul.

